# Lumbar spinal stenosis combined with obesity-induced idiopathic spinal epidural lipomatosis treated with posterior lumbar fusion: case report

**DOI:** 10.1186/s12893-021-01157-8

**Published:** 2021-04-26

**Authors:** Xiao Han, Derong Xu, ZhiNan Ren, Xin Chen, Zheng Li, Shugang Li

**Affiliations:** 1 Department of Orthopaedics, Medical College Hospital, Chinese Academy of Medical Sciences and Peking Union Medical College, Peking UnionDongcheng District Shuaifuyuan No. 1, Beijing, 100730 China; 2 Department of Orthopedics, The Affiliated Hospital of Qingdao University, Qingdao, China; 3 Department of Orthopaedics, The First Affiliated Hospital of Zhengzhou University, Zhengzhou, China

**Keywords:** Lumbar spinal stenosis, Obesity, Spinal epidural lipomatosis, Posterior lumbar fusion, Case report

## Abstract

**Background:**

Spinal epidural lipomatosis is a rare cause of lumbar spinal stenosis. While conservative therapy is applicable for most of cases, surgical intervention is necessary for severe ones. This is the first time we apply this modified technique to this disease.

**Case presentation:**

The case is a 53-year-old man. He is 175 cm tall and weighs 102 kg (body mass index 33.3 kg/cm^2^), presenting with low back pain and bilateral legs pain and numbness. Radiological examination showed severe lumbar spinal stenosis resulting from adipose hyperplasia, combined with hyperosteogeny and hypertrophy of ligaments, which are common etiological factors. Posterior decompression, internal fixation and a modified articular fusion technique was performed on this patient, and regular follow-up that up o 22 months showed outstanding clinical outcomes.

**Conclusions:**

A suitable style of posterior lumbar fusion should be considered to especially severe case with lumbar spinal stenosis and idiopathic spinal epidural lipomatosis.

## Background

Spinal epidural lipomatosis (SEL) is a rare disease featuring in overgrowth of epidural adipose tissue within the spinal canal [1]. Clinical categories depend on etiology, mainly including exogenous steroid use and endogenous steroid hormonal disease [2, 3]. But there is a disagreement about the relationship between idiopathic SEL and obesity. [4]. In our study, we prefer to classify obesity SEL as a special subtype of idiopathic SEL. As the disease progresses, surgical decompression turns out to be the final choice [6]. A series of studies have reported satisfactory results of decompression, internal fixation and bone graft fusion [7, 8].

Our medical group has been performing the posterior decompression, internal fixation and modified articular fusion to treat lumbar spinal stenosis for years and have observed excellent results. This article is about our first attempt to conduct this surgery on lumbar spinal stenosis (LSS) combined with obesity-related idiopathic SEL and it was proved to be an acceptable, feasible and clinically effective procedure for this rare disease.

## Case presentation

### History and imaging

A 53-year-old man with normal height (175 cm) and overweight (102 kg, body mass index = 33.3 kg/cm^2^) initially presented with low back pain and bilateral legs pain for 4 years and bilateral lower limps numbness for 2 years.

Four years ago, this patient gradually developed low back pain, radiating to hip and lateral side of both legs. The pain worsened after long time standing, walking and working and relieved when bending and crouching. Numerical rating scale (NRS, an 11-point scale where 0 = no pain and 10 = the worst pain) scored 4. The patient tried some unknown painkillers but they did not work. The intermittent pain lasted for years until 2 years ago when he started to have numbness of both lower limps, ranging from both sides of calfs to the dorsum of feet. Intermittent claudication occurs within 500 m’ walking. No cauda equina syndrome (CES) appeared. It should be noticed that this man gained 55 kg in weight during the past 20 years. Relevant history included hypertension for 10 years, smoking for 30 years and drinking for 30 years. In recent two weeks there was a fluctuation of blood sugar but no formal monitoring record was available. This patient had never received steroids medications.

**Fig. 1:**
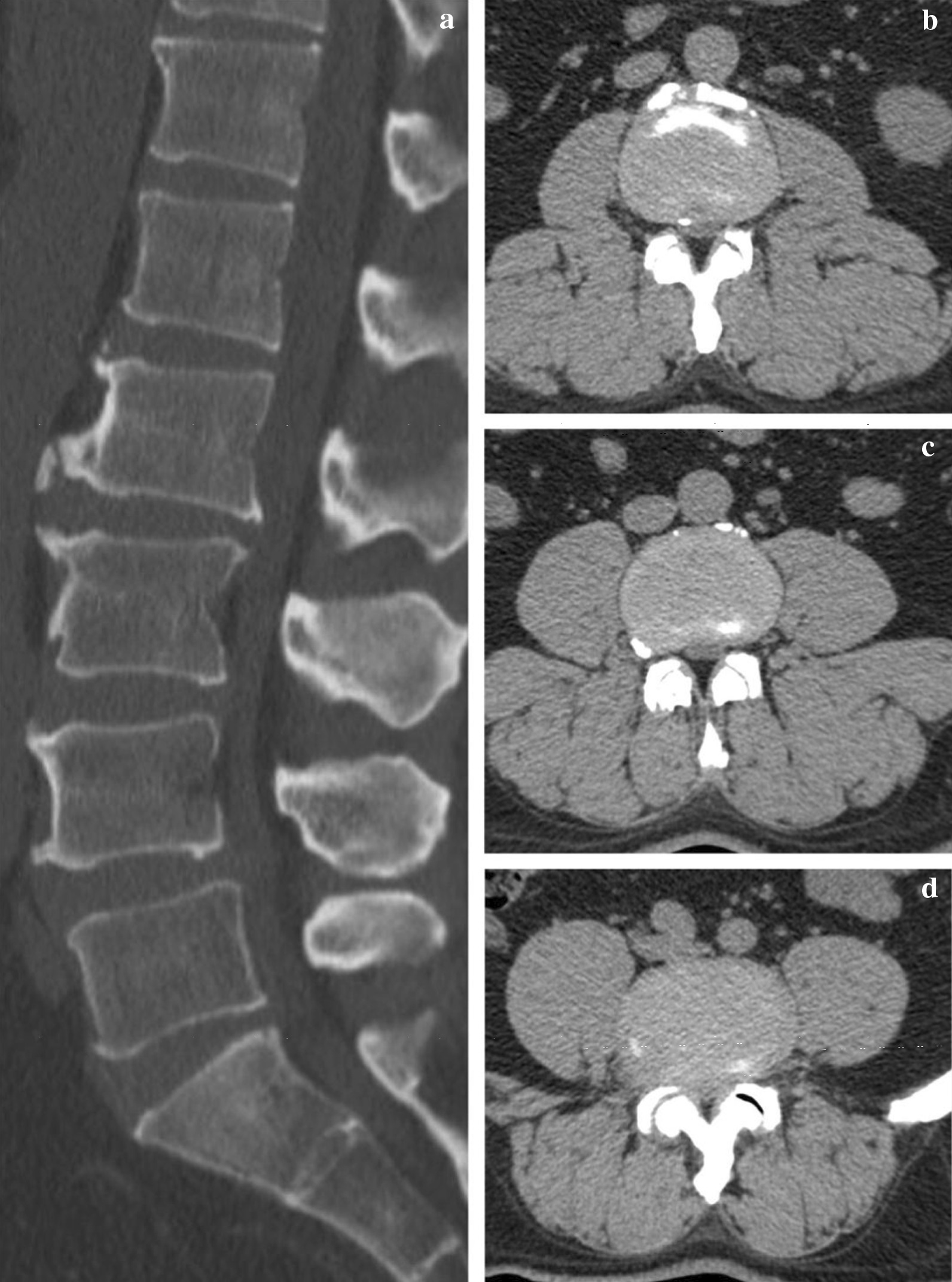
Preoperative CT scan. Preoperative sagittal CT scan indicates intervertebral space narrow, vertebral body bone hyperplasia, “Y” shape homogenious hypodense shadow and oval compression of caudal spinal cord. Axial images shows stenosis frome L2 to L5 and mild hypertrophy of ligamentum flavum and facet joint. **a** Sagittal image. **b** Axial image of L2/3. **c** Axial image of L3/4. **d** Axial image of L4/5

**Fig. 2:**
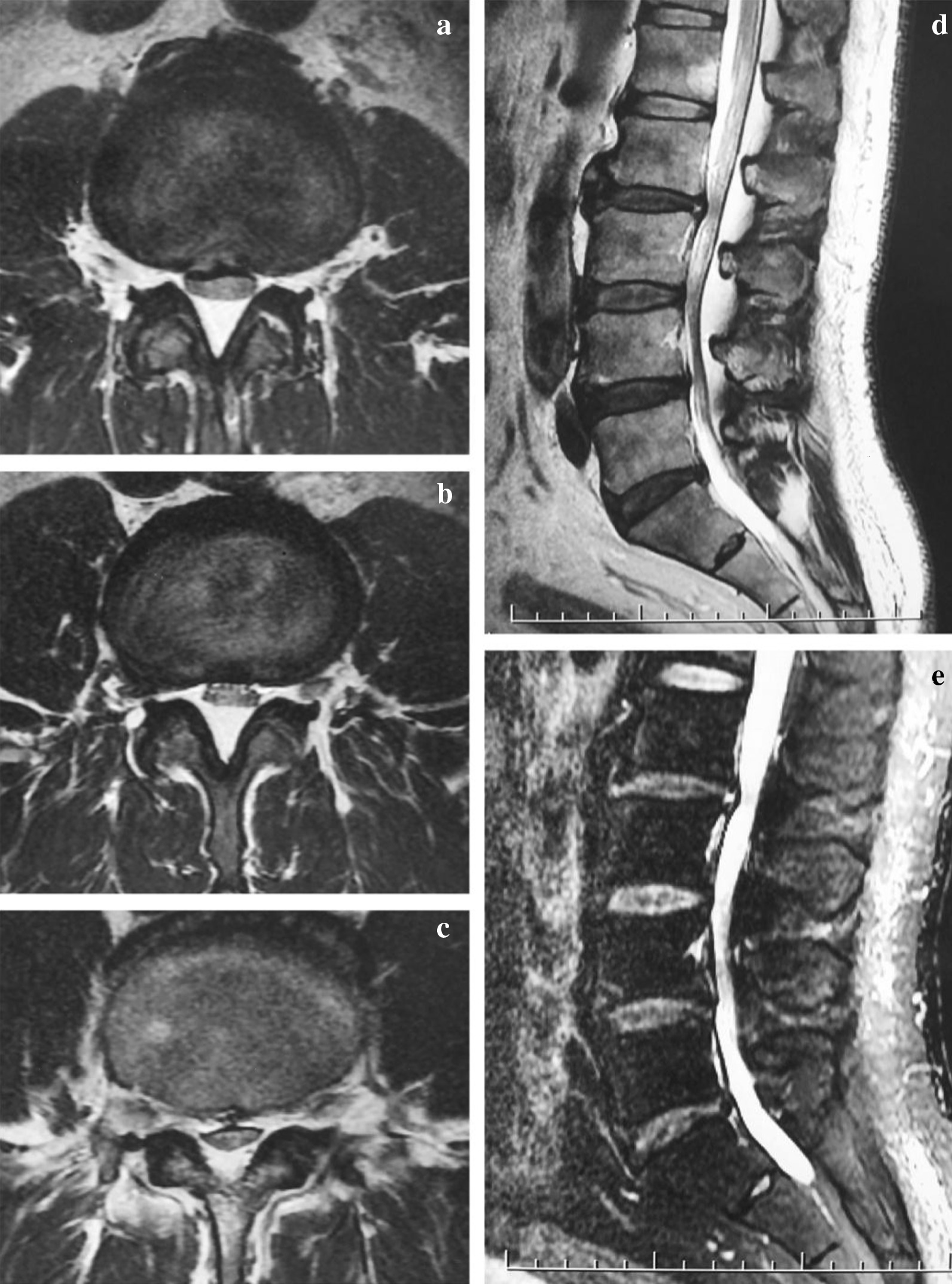
Preoperative MRI. T2-weighted axial images show classic “Y-sign” thecal compression secondary to epidural lipomatosis from L2-L5. **a** Axial image of L2/3. **b** Axial image of L3/4. **c** Axial image of L4/5. **d** T2-weighted sagittal image shows epidural lipomatosis as short homogeneous signal, compression and ventral shift spinal cord. **e** Fat suppression image shows suppression of adipose tissue

A full endocrinologic workup was conducted in patient to rule out underlying endocrine abnormalities and type 2 diabetes mellitus was diagnosed. On physical exam, the patient showed 5/5 strength, normal muscle tone and intact superficial sensation in the upper and lower extremities bilaterally. Tendon reflexes of the right knee was absent and several myelopathic signs were recorded as follows: Chaddock sign ( +) on the left, Oppenheim sign ( +) on the left and Babinski sign suspected ( +) on both sides. CT scan indicated typical signs of LSS: intervertebral foraminal stenosis, ligamentum flavum hypertrophy, disc paracentral herniation and spinal stenosis from L2 to L5 (Fig. 1), and MRI showed epidural abnormal fat deposition in the spinal canal from L2–L5 (Fig. 2). According to a previous study of Borre et al., SEL could be graded on basis of the degree of epidural adipose tissue occupying the spinal canal (extradural fat [EF]-to spinal canal [SC] ratio); grade I, 41–50% of the canal; grade II, 51–74% of the canal; and grade III, > 75% of the canal [9]. This patient’s imaging revealed grade II (60% EF/SC ratio) lumbar epidural lipomatosis (Fig. 2d).

Based on the patient’s symptoms, history, physical examination and radiological imaging, a primary diagnosis of LSS and obesity-induced idiopathic SEL from L2–L5 was proposed. The compression of the cauda equina came from anterior protruding lumbar disc and posterior adipose tissue and ligamentum flavum, and pressure on nerve root is caused by intervertebral foraminal bony stenosis. Since refractory radiculopathic symptoms had appeared, indication of surgery was clear. Mimimally invasive surgery like endoscopic technique was insufficient to relieve bony stenosis, so extensive decompression was necessary. Internal fixation and bone graft fusion were adopted to maintain the stability of responsible vertebrae. Although the modified articular fusion had never been reported in such a rare disease, it was performed successfully in a large amount of obese patients with lumbar degenerative disease only. This was our first application of this modified surgical style on obesity SEL.

### Surgical procedure

**Fig. 3:**
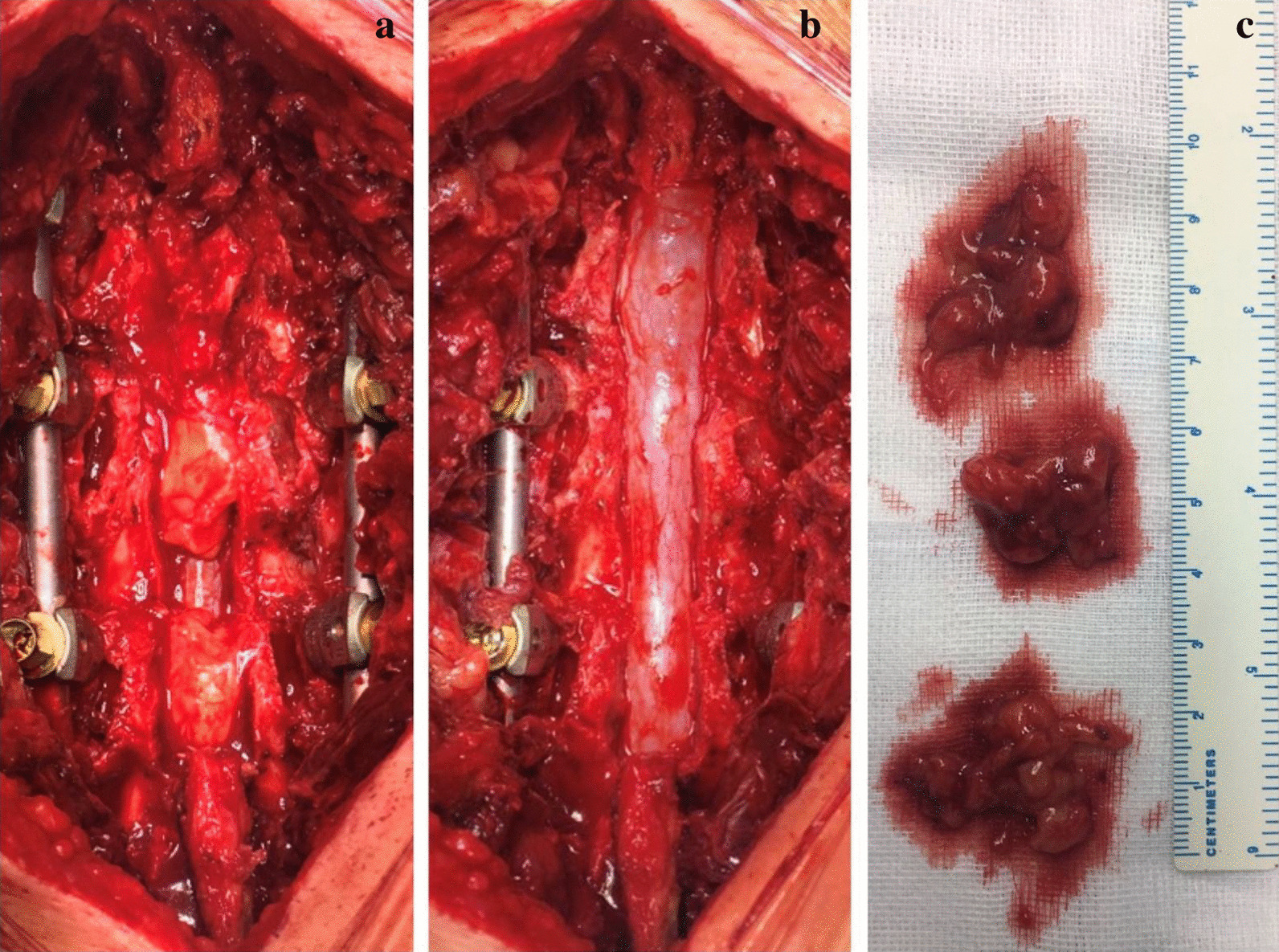
Intraoperative image. **a** Intraoperative image shows accumulation of epidural adipose tissue. **b** Decompression range includes bony structure and adipose tissue. **c** Resected adipose tissue with normal appearance

After a preoperative use of prophylactic antibiotics (cefuroxime 1.5 g, intravenous), the operation was performed under general anesthesia and in a prone position. Following careful stripping of the paraspinal muscles, spinous process, bilateral articular processes and roots of transverse processes were exposed. Afterwards, titanium polyaxial pedicle screws (6.5 mm in diameter, 50 mm long, Legacy, Medtronic, USA) were inserted bipedicularly from the L2 to L5 vertebrae. We chose used F4 reverse thread screws providing a tightening power in horizontal direction and minimizes the possibility of pulling out. This is quite important for such a patient with hight body weight. Then, pre-bended titanium rods were implanted and intraoperative fluoroscopy confirmed satisfactory position and length of screws. The resection range included spinous processes (L3, L4 and small portion of L2 and L5), 2/3 of the inferior L2 lamina, L3 and L4 lamina and 1/5 of upper L5 lamina, as well as the corresponding supraspinous ligament, interspinous ligament, ligamentum flavum and joint capsule. Then hyperplastic medial portion of facet joints, atrophic ligamentum flavum, stenosis of lumbar lateral recess and accumulation of epidural adipose tissue contributing to dural sac compression was visible (Fig. 3a). Decompression range of L2–L3 included medial 2/3 of the L2 bilateral inferior articular process, 1/3 of the medial side of bilateral superior articular processes of L5 and hyperplastic medial portion of L3 superior articular processes. Medial 2/3 of the bilateral L2 inferior articular process and 1/3 of the medial side of bilateral superior articular processes of L3 was carried out with a chisel and the lateral 2/3 portion was preserved for bone grafting. Management of L3–L4 and L4–L5 was the same as the above description. Following the decompression was resection of epidural adipose tissue with bipolar cautery (Fig. 3b).

Subsequently, inspection verified an adequate relaxation of nerve root and full decompression of dural sac. Removed tissue (Fig. 3c) had the appearance of normal adipose tissue. A round head grinding drill was then used to prepare the inter-articular space as grafting bed for autograft and allograft cancellous bone. After that, the surgery field was rinsed with saline before bone graft was pressed into the inter-articular space. Finally, a drainage tube was put in place and the wound was closed up. Intraoperative hemorrhage was 586 mL.

### Postoperative course

**Fig. 4:**
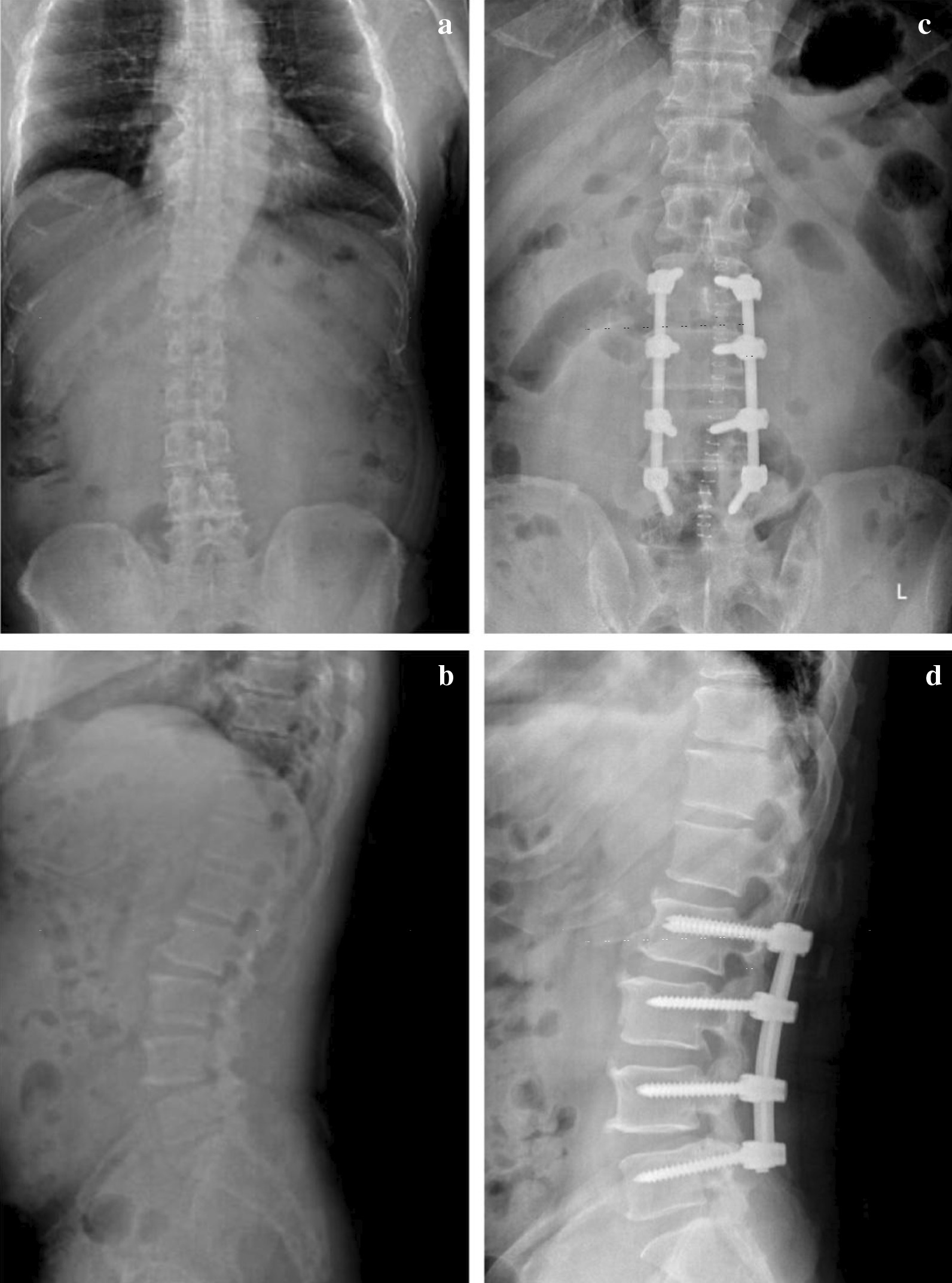
Preoperative and immediate postoperative X-Ray. Immediate postoperative X-Ray image in comparison of the preoperative counterparts shows satisfactory location of graft. **a** Preoperative coronal image. **b** Preoperative sagittal image. **c** Postoperative coronal image. **d** Postoperative sagittal image

**Fig. 5:**
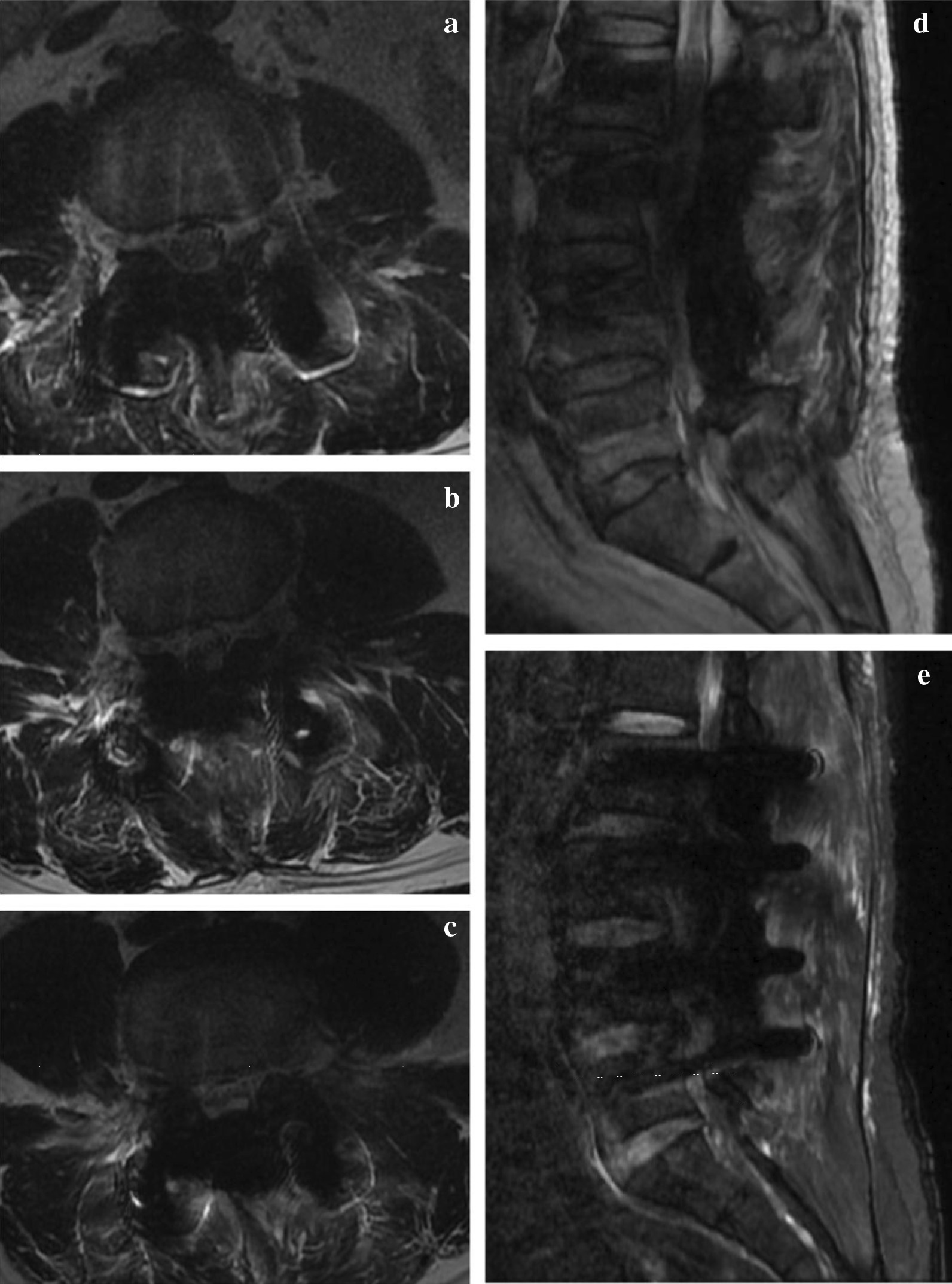
Immediate postoperative MRI. Immediate postoperative MRI image shows complete cancelation of extral adipose tissue and adequate decompression. **a** Axial image of L2/3. **b** Axial image of L3/4. **c** Axial image of L4/5. **d** T2-weighted sagittal image. **e** Fat suppression image

The patient showed full muscle strength and sensation once regained consciousness. Preoperative symptoms relieved obviously during the following days, and NRS scored 1. We removed the drainage tube and encouraged the patient to stand and walk on the third day after operation. Radiographs of lumbar spine were taken to verify the good location of screws and rods (Fig. 4), and MRI confirmed complete decompression and elimination of excessive fat tissue (Fig. 5). The patient was permitted to discharge with referral for outpatient rehabilitation. We also mobilized this patient to lose weight.

**Fig. 6:**
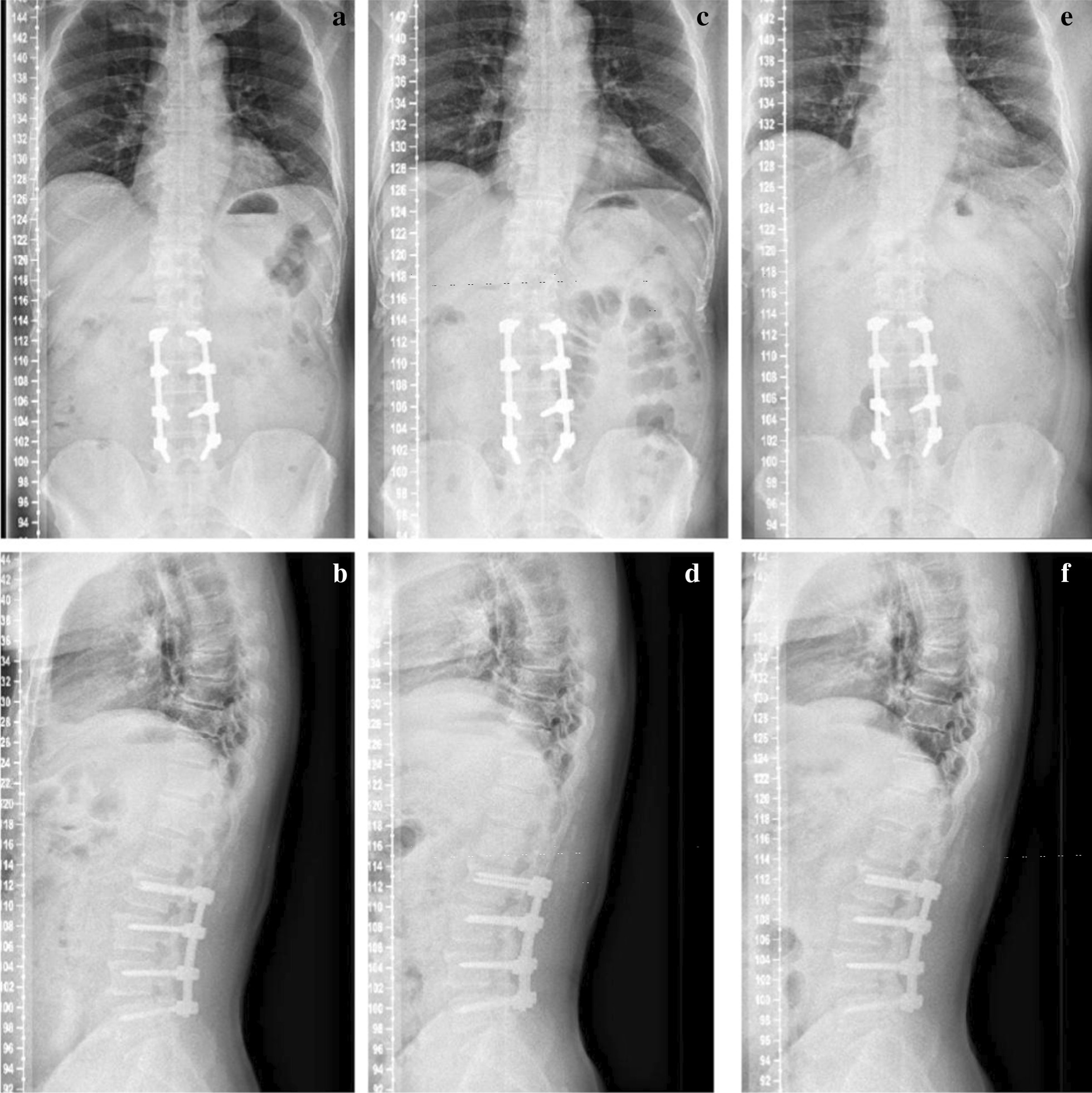
Follow-up X-Rays. Follow-up imaging examination shows consistent spinal stability and excellent internal fixation. **a** Coronal image of 3 months after surgery. **b** Sagittal image of 3 months after surgery. **c** Coronal image of 6 months after surgery. **d** Sagittal image of 6 months after surgery. **e** Coronal image of 22 months after surgery. **f** Sagittal image of 22 months after surgery

**Fig. 7:**
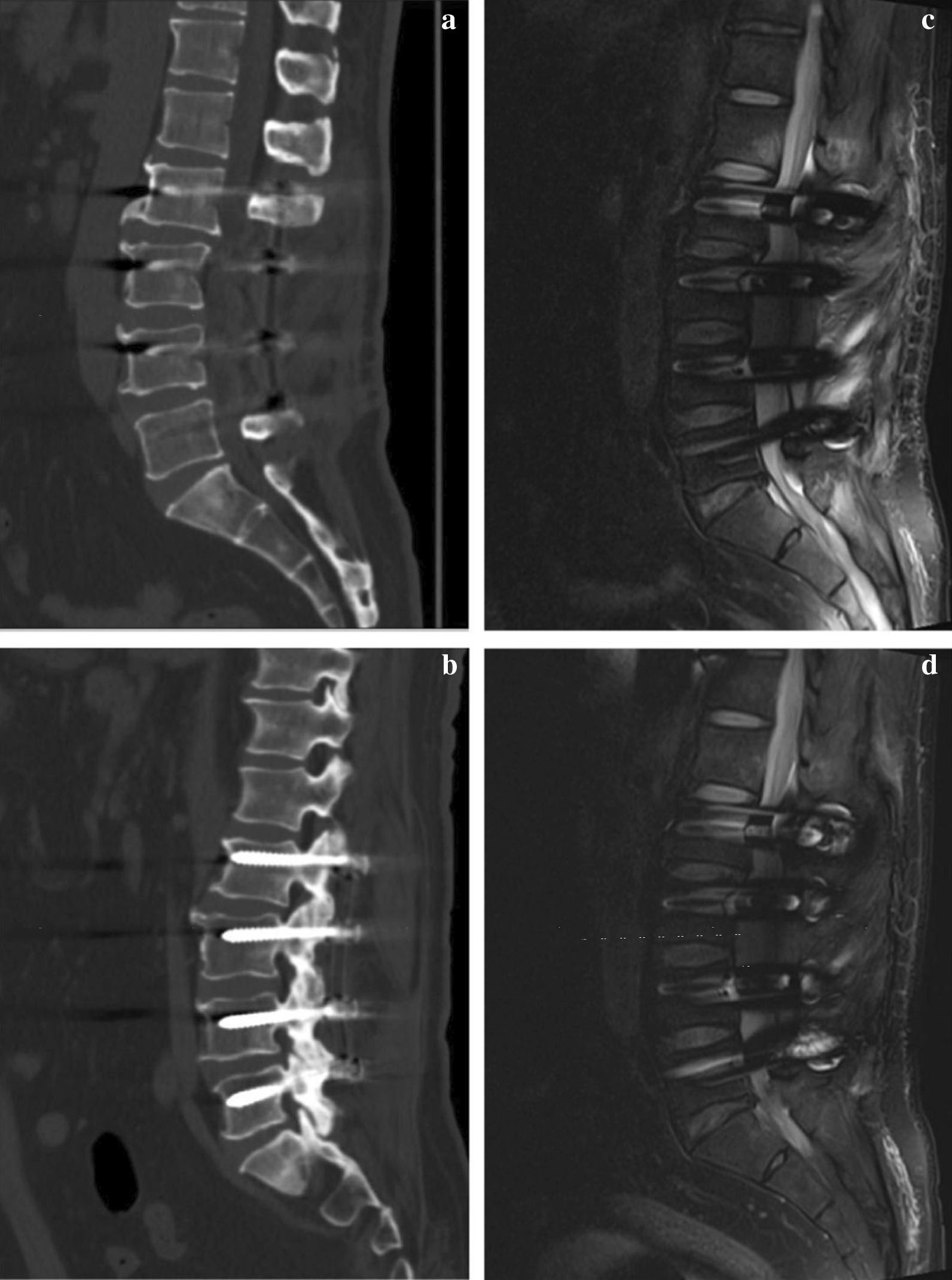
Postoperative CT scan and MRI. Postoperative CT scan shows satisfactory fusion rate of joint facet. MRI has not detected reoccurrence of spinal epidural lipomatosis. **a** Sagittal CT image of 6 months after surgery. **b** Sagittal CT image of 22 months after surgery. **c** Sagittal MRI image of 6 months after surgery. **d** Sagittal MRI image of 22 months after surgery

By now the man has come back for follow-up regularly and his low back and radiating leg pain has completely resolved, and NRS scored 0. X-Ray, CT and MRI at different follow-up time points showed persistent stability and graft and satisfactory fusion rate, and no evidence of reoccurred SEL was detected (Figs. 6 and 7).

## Discussion

Spinal epidural lipomatosis (SEL) is a relatively rare disease with excess deposition of adipose tissue in epidural space. Most of SEL result from long-time use of steroid medications, accounting for over half of the patients [10]. A series of endocrine diseases like cushing syndrome, hypothyroidism, carcinoid tumor, and pituitary prolactinoma have been proved to be risk factors of SEL [11, 12]. It has been reported that high level of steroid can lead to high levels of inflammatory cytokines [13], which results in overproduction of subcutaneous and visceral fat tissue [14], as well as epidural adipose tissue About 25% of SEL are associated with obesity [2], but not all of them can be classified into idiopathic SEL because some of them are correlated with definite endocrine disorders or use of steroid medications. The case we present has no history of steroid hormonal treatment or underlying related comorbidities, so we tend to group this in idiopathic SEL induced by obesity without known causes. It has been reported that the incidence of lumbosacral region SEL is increased in patients with high BMI and type 2 diabetes mellitus [13, 15], which corresponds with the features of our case since his BMI was 33.3 kg/cm^2^, much higher than the obesity cut-off (30 kg/cm^2^). Weight reduction is still believed to be the first-line treatment of isolated SEL associated with obesity, and surgeries are only be considered when conservative measures fail [16].

Since our patient had LSS and SEL simultaneously, both contributing to compression and triggering radiculopathic symptoms, surgical intervention was necessary. When told about prognosis and possible complications of the surgery, this patient showed strong willingness because he was eager to relive pain and improve his life quality. In previous studies, a large part of SEL patients show myelopathic or radiculopathic symptoms similar to that of LSS, but neither preoperative image nor intraoperative inspection reveal bony stenosis, and laminectomy with adipose tissue resection was reported to be effective [17, 18]. In our case, stenosis is caused by both hyperostosis and extral adipose tissue, so the surgical process included a standard posterior intraspinal decompression and resection of adipose tissue.

In the current mainstream treatments for the LSS, posterior lumbar fusion (PLF) and posterior /transforaminal lumbar interbody fusion (P/TLIF) are the most commonly used therapeutic methods. PLF is a traditional fusion technique and is performed on the interface of the bilateral transverse processes, and the lateral cortical bone of the articular processes is prepared as grafting bed. This procedure is feasible and widely applied, while has been criticized for low fusion rate [19]. P/TLIF differs from PLF mainly because it provides a stable connection in anterior, middle and posterior column, thus bring higher fusion rate. However, with deeper investigation to biomechanical change after lumbar surgeries in recent years, an extremely hard fusion is not recommended as it creates a long arm and causes high strength and low modulus, and all above may play a vital role in pull-out of screws, adjacent vertebral fracture (AVF) and failure of fixation [20]. As reported previously, articular fusion surgery focuses only on the posterior column structure and does not change the normal construction of the other two columns, so the vertebra’s physiological function can be well preserved [21]. The most important modification of our technique is the preparation of bone graft bed. Traditional technique uses osteotome to create bone graft surface, and we use grinding drill to modify the bone graft surface, providing a better situation for fusion. So our fusion technique is a relatively elastic and is proposed to have better fusion rate. SEL patients usually have higher BMI, which is a potential risk factor of AVF and failure, as it brings with stronger pressure on vertebral body and artificial graft. Therefore, such fusion technique may be a better choice for SEL patients.

This was the first time we applied intraspinal decompression, modified articular fusion and adipose resection in LSS patient with obesity-induced idiopathic SEL. Due to unavoidable low scientific evidence level of case report and limited cases, the reliability and safety of this technique remains to be tested, and the follow-up period is still insufficient to show the long-term effect. However, this surgery was feasible to relieve compression and effective to improve patient’s quality of life by the 22nd months after operation. It has potential as an effective treatment for such complex disease.

In conclusion, we recommend surgical decompression for patients diagnosed with LSS combined with SEL and present with severe neural symptoms. Decompression could solve pressure from both adipose tissue and osteal stenosis, and our modified fusion technique may be an ideal procedure for those with high BMI.

## Data Availability

The medical records and original image used during this study are available from the corresponding author on reasonable request.

## Abbreviations

LSS: Lumbar spinal stenosis
SEL: Spinal epidural lipomatosis
PLF: Posterior lumbar fusion
P/TLIF: Posterior /transforaminal lumbar interbody fusion

## References

[1] Fassett DR, Schmidt MH (2004). Spinal epidural lipomatosis: a review of its causes and recommendations for treatment. Neurosurg Focus.

[2] Fogel GR, Cunningham PY, Esses SI (2005). Spinal epidural lipomatosis: case reports, literature review and meta-analysis. Spine J.

[3] Kotilainen E, Hohenthal U, Karhu J, Kotilainen P (2006). Spinal epidural lipomatosis caused by corticosteroid treatment in ulcerative colitis. Eur J Intern Med.

[4] Kim K, Mendelis J, Cho W (2019). spinal epidural lipomatosis: a review of pathogenesis, characteristics, clinical presentation, and management. Glob Spine J.

[5] Lee M, Lekias J, Gubbay SS, Hurst PE (1975). Spinal cord compression by extradural fat after renal transplantation. Med J Aust.

[6] Haddad SF, Hitchon PW, Godersky JC (1991). Idiopathic and glucocorticoid-induced spinal epidural lipomatosis. J Neurosurg.

[7] Al-Khawaja D, Seex K, Eslick GD (2008). Spinal epidural lipomatosis—a brief review. J Clin Neurosci.

[8] Choi KC, Kang BU, Lee CD, Lee SH (2012). Rapid progression of spinal epidural lipomatosis. Eur Spine J.

[9] Borre DG, Borre GE, Aude F, Palmieri GN (2003). Lumbosacral epidural lipomatosis: MRI grading. Eur Radiol.

[10] Papastefan ST, Bhimani AD, Denyer S, Khan SR, Esfahani DR, Nikas DC, Mehta AI (2018). Management of idiopathic spinal epidural lipomatosis: a case report and review of the literature. Child's Nervous System ChNS.

[11] Bhatia K, Frydenberg E, Steel T, Ow-Yang M, Ho K, Grainger E (2010). Spinal epidural lipomatosis due to a bronchial ACTH-secreting carcinoid tumour. J Clin Neurosci.

[12] Koch CA, Doppman JL, Watson JC, Patronas NJ, Nieman LK (1999). Spinal epidural lipomatosis in a patient with the ectopic corticotropin syndrome. N Engl J Med.

[13] Fujita N, Hosogane N, Hikata T, Iwanami A, Watanabe K, Shiono Y, Okada E, Ishikawa M, Tsuji T, Shimoda M (2016). Potential involvement of obesity-associated chronic inflammation in the pathogenesis of idiopathic spinal epidural lipomatosis. Spine.

[14] Alicioglu B, Sarac A, Tokuc B (2008). Does abdominal obesity cause increase in the amount of epidural fat?. Eur Spine J.

[15] Yildirim B, Puvanesarajah V, An HS, Novicoff WM, Jain A, Shen FH, Hassanzadeh H (2016). Lumbosacral epidural lipomatosis: a retrospective matched case-control database study. World Neurosurg.

[16] Moller JC, Cron RQ, Young DW, Girschick HJ, Levy DM, Sherry DD, Kukita A, Saijo K, Pessler F (2011). Corticosteroid-induced spinal epidural lipomatosis in the pediatric age group: report of a new case and updated analysis of the literature. Pediatr Rheumatol Online J.

[17] Ferlic PW, Mannion AF, Jeszenszky D, Porchet F, Fekete TF, Kleinstuck F, Haschtmann D (2016). Patient-reported outcome of surgical treatment for lumbar spinal epidural lipomatosis. Spine J.

[18] Kellett CG, Siva V, Norman ICF, Jung J, Grahovac G, Minhas P (2019). Symptomatic idiopathic spinal epidural lipomatosis in 9 patients: clinical, radiologic, and pathogenetic features. World Neurosurg.

[19] Levin JM, Tanenbaum JE, Steinmetz MP, Mroz TE, Overley SC (2018). Posterolateral fusion (PLF) versus transforaminal lumbar interbody fusion (TLIF) for spondylolisthesis: a systematic review and meta-analysis. Spine J.

[20] Li YC, Yang SC, Chen HS, Kao YH, Tu YK (2015). Impact of lumbar instrumented circumferential fusion on the development of adjacent vertebral compression fracture. Bone Jt J.

[21] Hrabálek L, Wanek T, Adamus M, Cecháková E, Buřval S, Langová K, Vaverka M (2014). Surgery for degenerative spondylolisthesis of the lumbar spine using intra-articular fusion. A prospective study. Acta Chirurgiae Orthopaedicae Traumatologiae Cechoslovaca.

